# Beyond NF-κB activation: nuclear functions of IκB kinase α

**DOI:** 10.1186/1423-0127-20-3

**Published:** 2013-01-23

**Authors:** Wei-Chien Huang, Mien-Chie Hung

**Affiliations:** 1 Center for Molecular Medicine, China Medical University Hospital, Taichung, 40447, Taiwan; 2 Graduate Institute of Cancer Biology, China Medical University, No.6 Hsueh-Hsih Road, Taichung, 404, Taiwan; 3 The Ph.D. program for Cancer Biology and Drug Discovery, China Medical University, Taichung, 404, Taiwan; 4 Department of Biotechnology, Asia University, Taichung, 413, Taiwan; 5 Department of Molecular and Cellular Oncology, The University of Texas MD Anderson Cancer Center, Unit 108, 1515 Holcombe Blvd, Houston, Texas, 77030, USA

**Keywords:** Nuclear IKKα, NF-κB, Gene transcription, Tumor progression

## Abstract

IκB kinase (IKK) complex, the master kinase for NF-κB activation, contains two kinase subunits, IKKα and IKKβ. In addition to mediating NF-κB signaling by phosphorylating IκB proteins during inflammatory and immune responses, the activation of the IKK complex also responds to various stimuli to regulate diverse functions independently of NF-κB. Although these two kinases share structural and biochemical similarities, different sub-cellular localization and phosphorylation targets between IKKα and IKKβ account for their distinct physiological and pathological roles. While IKKβ is predominantly cytoplasmic, IKKα has been found to shuttle between the cytoplasm and the nucleus. The nuclear-specific roles of IKKα have brought increasing complexity to its biological function. This review highlights major advances in the studies of the nuclear functions of IKKα and the mechanisms of IKKα nuclear translocation. Understanding the nuclear activity is essential for targeting IKKα for therapeutics.

## Introduction

IκB kinase (IKK)/Nuclear factor kappa B (NF-κB) family signaling mediates the expression of hundreds of genes involved in inflammation, immune response, cell survival, and cancer [1-4]. NF-κB proteins are part of a molecular cascade that begins with signals outside the cell and culminates in the nucleus by binding to DNA and activating gene expression. The best-known form of NF-κB consists of the DNA-binding subunit p50 and a transcription activator, p65 (also known as Rel A). In the absence of specific extracellular signals, NF-κB inhibitors, such as IκB, p105, and p100 proteins, tether to NF-κB in the cytoplasm to prevent NF-κB-mediated gene transcription [1]. When cells receive appropriate stimuli, such as tumor-necrosis factor-α (TNF-α), a ternary IKK complex consisting of IKKα, IKKβ and NEMO (IKKγ) induces IκB phosphorylation, leading to IκB ubiquitination and proteasomal degradation that are required for liberation of NF-κB in the nucleus where it binds to specific promoter elements to activate gene expression [1,5].

Although IKKα and IKKβ share structural and biochemical similarities, different phenotypes between IKKα and IKKβ knockout mice imply distinct physiological roles of the IKK isoforms [6]. IKKβ deficiency results in embryonal death and shows the defective response to inflammatory cytokines and liver cell apoptosis [7]. IKKα knockout mice display the defective proliferation and differentiation of kerationocyte and the abnormalities of limb and skeleton, suggesting the requirement of the IKKα subunit in morphogenesis [8]. Importantly, these studies of gene knockout have shown that IKKα is dispensable for IκB degradation although both IKKα and IKKβ are critical for NF-κB-mediated gene expressions. Instead of its role in phosphorylating IκBα in classic NF-κB activation, IKKα homodimer has been shown to mediate the processing of p100 precursor to p52 by the noncanonical NF-κB pathway [5]. IKKα- and IKKβ-deficient mouse embryo fibroblasts exhibit different patterns of β-catenin cellular localization in which IKKβ decreases β-catenin-dependent transcriptional activation while IKKα increases β-catenin-dependent transcriptional activity [9]. Differential requirements for IKKα and IKKβ were also found in primary human osteoarthritic chondrocyte differentiation [10].

In addition to phosphorylating distinct substrates in the cytoplasm [6,11], sub-cellular distribution of IKKα is also different from that of IKKβ, further indicating that these two related signaling kinases are functionally different. Many studies have indicated that IKKα can be detected in both the cytoplasm and the nucleus whereas IKKβ is detected predominantly in the cytoplasm [12-16]. The observation of nuclear/cytoplasm shuttling of IKKα led to the discovery of the first nuclear role of IKKα in phosphorylating histone H3, which results in NF-κB-mediated gene expression [12,16]. These studies provided an explanation of why IKKα is dispensable for IκBα degradation but remains essential for NF-κB-dependent transcription. Aside from nuclear regulation of NF-κB-dependent gene transcription through chromatin modification in response to pro-inflammatory stimuli, nuclear IKKα also functions in apoptosis, cell cycle, and tumor progression in colorectal [17,18], breast [19,20], pancreatic [21], gastric [22], osteosarcoma [23], and prostate [24] cancers. The current understandings of nuclear translocation and functions of IKKα are discussed below.

### Nuclear IKKα regulates NF-κB-dependent gene transcription and inflammation

Nuclear expression and functions of IKKα were first discovered based on the observation of different patterns of β-catenin activation in IKKα- and IKKβ-deficient mouse embryonic fibroblast (MEF) cells by Lamberti *et al.* in 2001 [9]. Their study showed β-catenin-dependent transcription was decreased by IKKβ but increased by IKKα that is likely due to the differential abilities of IKKα and IKKβ to bind to and phosphorylate β-catenin. Unlike the predominantly cytoplasmic IKKβ, IKKα has been detected in both the nucleus and the cytoplasm of MEF cells at resting state [9,25]. The constitutive shuttling of IKKα between cytoplasm and nucleus was further confirmed by accumulation of IKKα in the nucleus of Hela cells in the presence of a nuclear export blocking agent, leptomycin B (LMB) [13]. Therefore, the protein binding and phosphorylation of specific pools of β-catenin by IKKα in the nucleus have been proposed to explain the contradictory effect of the IKK isoforms on β-catenin-dependent transcription [9]. However, the phosphorylation sites and detailed mechanisms that account for the differential regulation of β-catenin by IKK isoforms remain to be explored.

**Figure 1:**
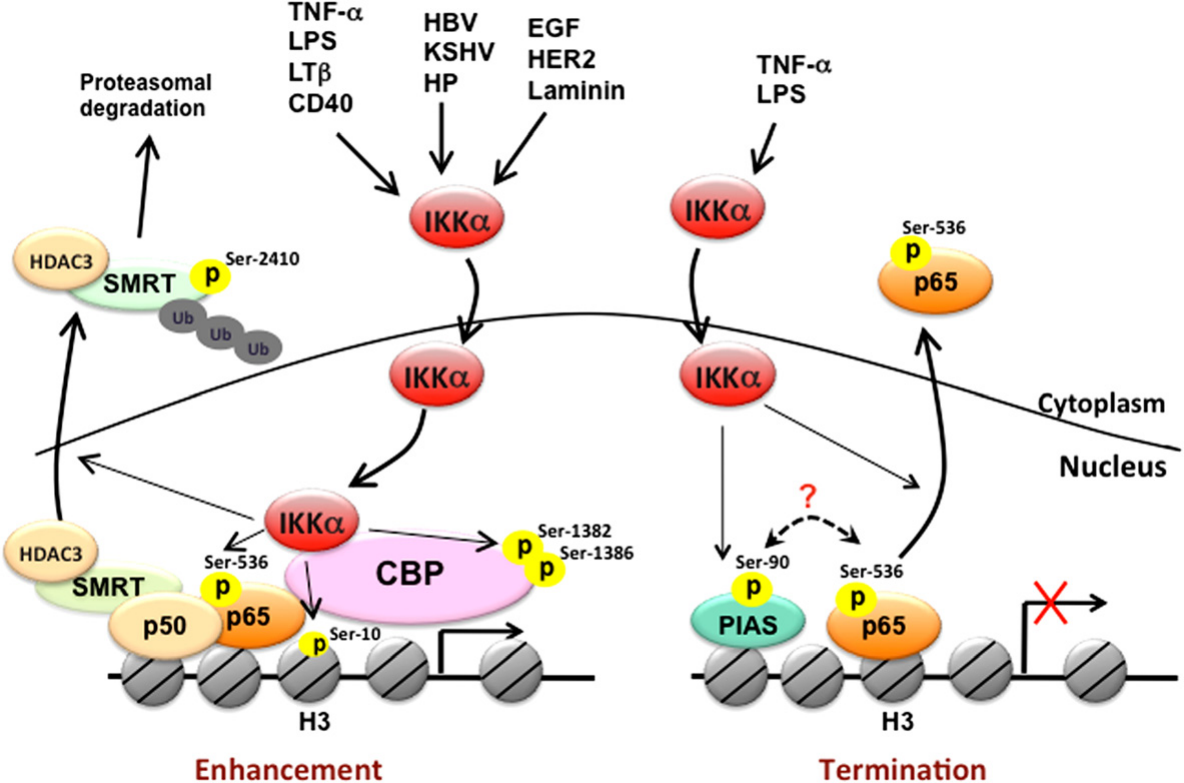
**Nuclear IKKα-dependent molecular regulations of NF-κB-mediated gene transcription.** In response to a variety of stimuli, including proinflammatory cytokines, pathogens, and growth factors, IKKα translocates into the nucleus and bind to DNA in association with CBP to phosphorylate histone H3 at Ser10, CBP at Ser1382/1386, and p65 at S536. Nuclear IKKα also removes repressive HDAC3/SMRT complex from NF-κB-dependent gene expression through phosphorylating SMRT at Ser2410. These events facilitate the formation of transcriptional enhanceosome to increase NF-κB-dependent gene expression. On the other hand, nuclear IKKα also contributes to the termination of NF-κB-mediated gene transcriptions by phosphorylating p65 at Ser536 and PIAS at Ser90 to facilitate the turnover of p65 in response to TNF-α or LPS stimulation.

IKK complex is activated in response to various stimuli involving inflammation, apoptosis, immune response, and cancer. These physiological and pathological stimuli have been reported to enhance the nuclear levels of IKKα (Figure 1). TNF-α, a critical pro-inflammatory cytokine, was the first stimulant for IKKα nuclear translocation identified [12,16]. Other inducers of alterative NF-κB pathway, including lymphotoxin β and CD40, also elicit nuclear IKKα signaling [15]. In addition to TNF-α, *Helicobacter pylori* (*HP*) can also trigger nuclear translocation of IKKα via its virulence factor CagA protein to induce cytokine production for appropriate inflammatory responses [22]. Similarly, overexpression of Kaposi’s sarcoma-associated herpesvirus (KSHV)-encoded viral FLICE inhibitory protein K13 [26] and hepatitis B virus-encoded X (HBx) protein [14] also induce IKKα nuclear translocation to regulate NF-κB activity. The requirement of cytoplasmic/nuclear shuttling and chromatin association of IKKα for NF-κB-dependent gene regulation in a TAK1-dependent manner in TLR4-activated antigen-presenting cells [27] and activated neutrophils [28,29] further support that nuclear IKKα functions as a common and important regulator for NF-κB activity in response to various immune and inflammatory stimuli (Figure 1).

As illustrated in Figure 1, TNF-α-induced nuclear IKKα mediates NF-κB-dependent gene transcription, regardless of IκBα degradation, by enhancing transactivation [25] and DNA binding [30] of p65 as well as chromatin regulation through its interaction CREB-binding protein (CBP), a histone acetyltransferase [15,16]. Phosphorylation of serine residues within the transactivation domains (TA1 and TA2) of p65 is responsible for transcriptional activation of the NF-κB target genes [31]. By employing the Gal4 DNA binding domain fused to the p65 transactivation portion in a heterologous luciferase reporter assay, nuclear IKKα was shown to transduce NF-κB-inducing kinase (NIK)-dependent p65 TA1 transcriptional activity, suggesting that IKKα phosphorylates the TA1 domain of p65 in the nucleus [25]. In addition, using the IKKα mutant lacking an intact nuclear localization sequence (NLS), p65 chromatin immunoprecipitation assays further revealed that nuclear IKKα plays a role in binding activity of NF-κB/p65 to some but not all NF-κB-target promoters by removing histone deacetylase 3 (HDAC3), a negative regulator of NF-κB-dependent transcription, from these promoters [30]. Even though several earlier studies have implicated the function of IKKα in chromatin, the role of nuclear IKKα as a chromatin modifier was not reported until later by Baldwin’s and Gaynor’s group [12,16]. They showed that IKKα functions as a chromatin kinase in the nucleus and targets histone H3 at Ser10 for activation of NF-κB-directed gene expression (Figure 1). The nuclear import, chromatin association, histone phosphorylation of IKKα relies on its kinase activation by the upstream NIK kinase in response to both TNF-α and endotoxin lipopolysarccharide (LPS) [32]. However, the correlation between chromatin-bound IKKα and histone H3 phosphorylation on NF-κB-target genes was not found in human prostate carcinoma DU145 cells [18], indicating that cell type- and target gene-specificity exists in IKKα-dependent histone phosphorylation.

Gaynor’s group further showed that nuclear IKKα interacts with the transactivation domain of CBP. The IKKα/CBP complex in conjunction with p65 is recruited to the NF-κB responsive promoters to mediate cytokine-induced phosphorylation and subsequent acetylation of specific residues in histone H3 [16]. These findings suggested that IKKα not only targets on NF-κB but also functions as a key epigenetic regulator to initiate sequential chromatin modifications. Our studies also demonstrated that nuclear IKKα binds directly to CBP and phosphorylates its HAT domain at Ser1382 and Ser1386 to enhance the enzymatic activity of CBP on histone acetylation [15] (Figure 1). CBP and its homolog, p300, are transcriptional coactivators that function in the communication between transcription factors and the transcriptional machinery [33]. Since the availability of these coactivators is limited due to their essentiality for large number of transcription factors, competition between different transcription factors for CBP or p300 has been proposed to play a role in the coordination of gene expression and the appropriate execution of many biological processes [34]. Our study further demonstrated that the IKKα-dependent CBP phosphorylation enhances NF-κB-mediated gene expression and suppresses p53-mediated gene expression by switching the binding preference of CBP from p53 to NF-κB, thereby promoting cell growth [15]. Similar to IKKα, IKKγ/NEMO has also been shown to shuttle between the cytoplasm and the nucleus and to compete with p65 and IKKα for binding to the N-terminus of CBP. Even though IKKγ/NEMO is essential for the kinase activity of IKK complex in the cytoplasm, it seems to act as a negative regulator of nuclear IKKα and inhibit CBP-dependent transcriptional activation in the nucleus [35].

CBP and p300 mediate acetylation of histones as well as many transcription factors, including p65, for their transcriptional potential [33]. Acetylation of p65 is critical for its DNA binding and transactivation activity [36,37]. In contrast, deacetylation of p65 by HDACs, including HDAC1, HDAC2, HDAC3, and SIRT1, has been reported to repress its transcriptional activity [38,39]. The enzymatic activities of HDACs are regulated by their ability to associate with co-repressor proteins, such as SMRT and NCoR [40]. Chromatin-associated HDAC3/SMRT complex tethered by p50 homodimers on NF-κB-target *cIAP* and *IL-8* promoters of unstimulated cells is responsible for the basal suppression of NF-κB-regulated gene transcriptions [18]. In addition to enhancing histone acetylation by targeting CBP, IKKα was also found to relieve the suppression of NF-κB-mediated transcription by removing the HDAC3/SMRT repressor complex from target promoters [18,30,41]. Chromatin immunoprecipitation analysis demonstrated that upon attachment to laminin, the induction of chromatin-associated IKKα protein and acetylated histone is accompanied by a decrease in chromatin-bound HDAC3/SMRT complex. Direct phosphorylation of SMRT at Ser2410 by IKKα on chromatin also stimulates nuclear export and proteasomal degradation of the HDAC3/SMRT complex by recruiting TBL1/TBLR1, Ubc5, and 14-13-3ε proteins [18]. The removal of HDAC3/SMRT by IKKα allows the active p50-RelA/p65 heterodimer of NF-κB to bind and potentiate transcription (Figure 1). Although the SMRT corepressor returns to the chromatin-bound NF-κB complex almost immediately after the active p50-RelA/p65 heterodimer binds to the promoter, IKKα remains associated with the chromatin and phosphorylates both p65 at Ser536 and SMRT at Ser2410 to prevent the recruitment and chromatin association of HDAC3 at the NF-κB-regulated promoter. Displacement of HDAC3 from active NF-κB allows p300 to load and subsequently acetylate p65 at Lys310, which is required for full NF-κB transcription [41]. Thus, IKKα-mediated derepression of SMRT is an initial step critical for NF-κB transcription and survival in response to laminin attachment. Interestingly, cigarette smoke extract (CSE) was recently reported to induce the translocation of IKKα from cytoplasm to nucleus in mouse lung tissue in a dose-dependent manner. CSE-activated nuclear IKKα mediates the pro-inflammatory gene transcription through phospho-acetylation of RelA/p65 and histone H3 [42], suggesting nuclear IKKα-targeted histone H3, SMRT, and CBP/p300 play a role in CSE-induced NF-κB activation.

In contrast to activating NF-κB in response to proinflammatory stimuli, IKKα kinase activity has been reported to be required for the termination of NF-κB activation [43]. In response to the systemic challenge with the Gram-positive human pathogen group B *Streptococcus* (GBS), transgenic mice expressing the inactivatable variant of IKKα (AA) showed higher bacterial clearance but accelerated mortality compared with the wild-type mice. The exacerbated inflammatory phenotype was believed to be associated with this paradoxical result. Indeed, after administration of the *Escherichia coli*-derived LPS, transcripts of several pro-inflammatory and antiapoptotic NF-κB-target genes were higher in IKKα^AA^/^AA^ mice than in the littermate controls, indicating a role of IKKα in terminating the activation of classical NF-κB pathway in response to LPS-induced Toll-like receptor (TLR) signaling. It was then demonstrated that IKKα activity is required to accelerate the removal of RelA/p65 and c-Rel from pro-inflammatory gene promoters and the turnover of these NF-κB subunits by specifically phosphorylating p65 within its transactivation domain at Ser536. These events terminate LPS-induced NF-κB activation, leading to the negative regulation of macrophage activation and inflammation [43] (Figure 1). While much attention has focused on pro-inflammatory signaling, this study explored an opposing but complimentary role of IKKα in resolving inflammation. Since IKKα also showed nuclear function in histone phosphorylation in LPS-treated macrophage [32], this raises the possibility that LPS-activated IKKα also phosphorylates p65 at Ser536 in the nucleus to terminate the transcriptional activity of NF-κB. However, this hypothesis seems contradictory to the previous finding that TNF-α-induced NIK/IKKα complex phosphorylates p65 at Ser536 in the nucleus to enhance NF-κB activity [25]. These findings suggest that the transcriptional activity of NF-κB is not determined merely by p65 Ser536 phosphorylation and other regulatory factors are required. The work by Liu *et al*. further demonstrated that protein inhibitor of activated STAT1 (PIAS1), a gene-specific transcriptional repressor with SUMO E3 ligase activity, is involved in the IKKα-mediated negative regulation of NF-κB and inflammation restriction [44]. In response to various inflammatory stimuli, including TNF-α and LPS, PIAS is rapidly phosphorylated at Ser90, and this phosphorylation is mediated by IKKα and required for its association with chromatin and enzymatic activity to repress promoter-binding and transcriptional activities of NF-κB [44]. It would be interesting to further address the relationship between p65 Ser536 phosphorylation and PIAS Ser90 phosphorylation in the IKKα-mediated negative regulation of NF-κB activity (Figure 1).

### Nuclear functions of IKKα in NF-κB-independent gene transcription regulation

**Figure 2:**
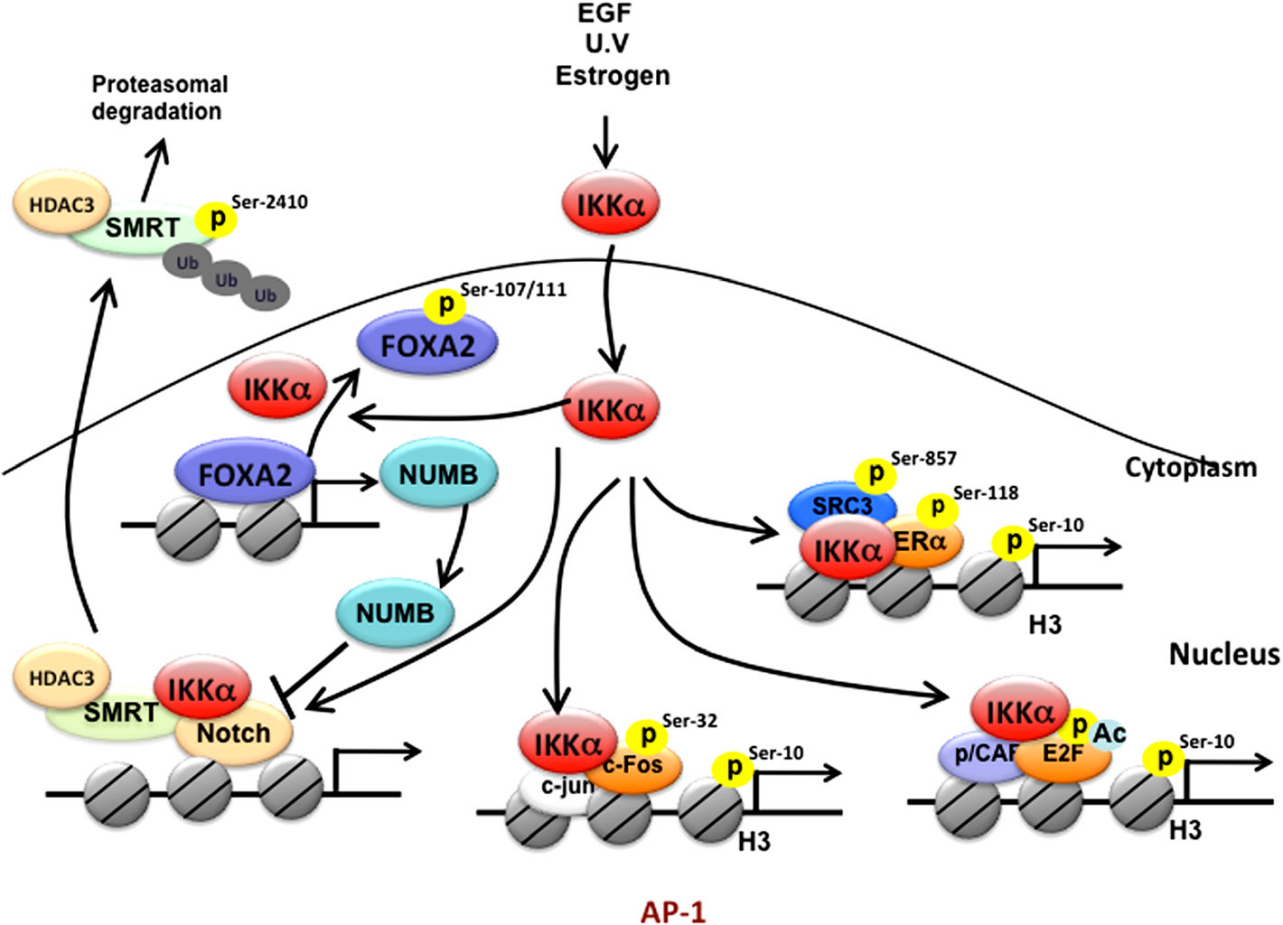
**The roles of nuclear IKKα in the regulation of NF-κB-independent gene transcription.** Nuclear IKKα enhances Notch-dependent gene transcriptional by phosphorylating and removing co-repressor SMRT from target gene promoters. IKKα also contributes to Notch transcriptional activity through phosphorylating and inactivating FOXA2, which subsequently leads to NUMB suppression. By direct target on transcription factors, nuclear IKKα also increases AP-1, ERα, and E2F-mediated gene transcription. Phosphorylation of SRC3 at Ser857 by nuclear IKKα also contributes to ERα transcriptional activity.

Nuclear IKKα-mediated histone H3 phosphorylation is involved in c-fos upregulation in a NF-κB-independent manner in response to EGF [45] and UV [46] stimulation (Figure 2). Dong *et al.* demonstrated that Ser32 phosphorylation, which is required for c-Fos protein stability and promoter recruitment of c-Fos, requires the kinase activity of nuclear IKKα [46], indicating that the chromatin regulation by nuclear IKKα is not limited to NF-κB-targeted genes but also affects gene transcriptions regulated through targeting various transcription factors or cofactors (Figure 2). For example, IKKα-mediated suppression of SMRT is required not only for NF-κB activation [18] but also Notch-dependent transcription [17], implying that nuclear IKKα may function as a common epigenetic regulator for gene transcription. Our recent study further indicated that nuclear IKKα may also derepress Notch-dependent transcription by diminishing the gene expression of NUMB [47], which targets Notch1 for lysosomal degradation through protein-protein interaction [48]. We identified forkhead box protein A2 (FOXA2) as the transcription factor for NUMB gene transcription and demonstrated that IKKα reduces the transcriptional activity of FOXA2 via binding to and phosphorylating it at Ser107 and Ser111 [47]. Therefore, nuclear IKKα may also enhance Notch-dependent gene transcription by suppressing FOXA2/NUMB signaling pathway (Figure 2). In addition to Notch, IKKα but not IKKβ was found to be involved in estrogen receptor (ER)-mediated gene transcription by binding to the estrogen-responsive elements (EREs)-containing promoters and phosphorylating histone H3, ERα, or coactivators such as AIB1/SRC-3. Coordinated promoter recruitment of ERs and specific coactivators, such as SRC-1, AIB1/SRC-3, GRIP1, CBP/p300, PCAF, CARM1, and PRMT1, is required for estrogen-regulated transcriptional activation. By forming a transcriptional complex with ERα and AIB/SRC3, IKKα mediates histone H3 phosphorylation on the promoters of several estrogen-responsive genes. In addition, nuclear IKKα also enhances the activities of ERα and AIB1/SRC-3 through phosphorylation of their Ser118 and Ser857 respectively (Figure 2), leading to the increase in cyclin D1 and Myc expression and estrogen-mediated breast cancer cell growth [19]. Since IKKα-activated SRC3 is also required for transcriptional activity of NF-κB [49], SRC-3 coactivator, in addition to histone H3 and SMRT, may also be another target of nuclear IKKα to increase general gene transcriptions.

In response to estrogen stimulation, IKKα also regulates cell cycle progression through modulating E2F1-depedent transcription [20]. The Rb/E2F pathway controls G1/S phase transition by activating expression of genes required for DNA replication. Silencing of IKKα but not IKKβ significantly reduced estrogen-induced cell cycle progression and transcription of the E2F1 gene as well as other E2F1-responsive genes, including thymidine kinase 1, proliferating cell nuclear antigen, cyclin E, and cdc25A, indicating that IKKα plays a critical role in regulating E2F-dependent gene transcription. Through association with E2F1, IKKα is recruited to E2F-1 responsive promoters and potentiates the ability of p300/CBP-associated factor to acetylate E2F1 for transcriptional activation in response to estrogen treatment (Figure 2). These findings suggest that nuclear IKKα influences estrogen-mediated cell cycle progression by modulating E2F1 at both the transcriptional and posttranscriptional levels [20]. Taken together, IKKα appears to modulate various epigenetic signaling pathways to regulate specific sets of genes.

### Regulation of apoptosis by nuclear IKKα

**Figure 3:**
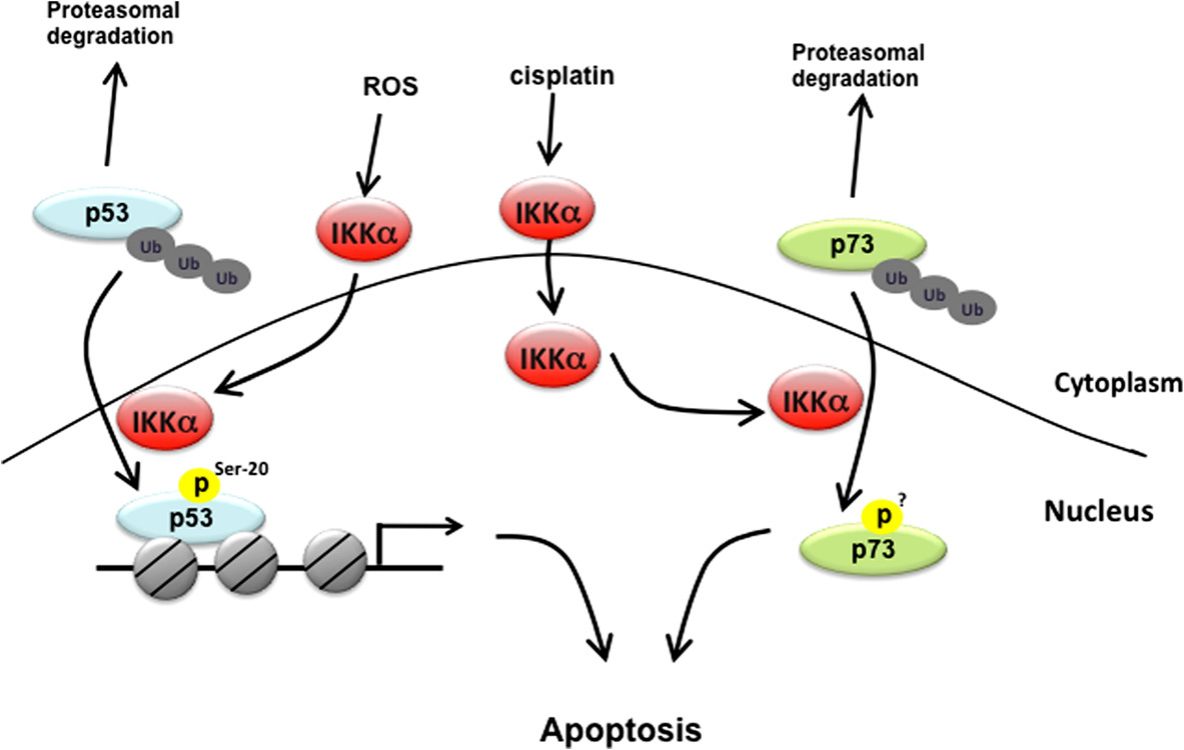
**Nuclear IKKα targets p53 and p73 to mediate apoptosis.** In response to DNA damage induced by ROS and cisplatin, nuclear IKKα stabilizes p53 and p73 protein level respectively to promote apoptosis.

NF-κB is activated to control apoptosis upon exposure to various cytotoxic stimuli, including reactive oxygen species (ROS). Recent evidence suggests a negative regulatory role of activated NF-κB in ROS-elicited JNK signaling to antagonize apoptosis [50]. Interestingly, the Src-dependent Tyr42 phosphorylation but not IKK-mediated Ser32/36 phosphorylation of IκBα contributes to ROS-induced NF-κB activation in a proteolysis-independent mechanism [51,52]. Although hydrogen peroxide stimulation has been shown to induce IKKα activity and thus its nuclear translocation, it had no effect on NF-κB activation [53], suggesting that IKKα is not involved in the ROS-induced NF-κB activation. In contrast to the pro-survival role of NF-κB, nuclear IKKα plays an opposite function in ROS-mediated apoptosis through modulating p53 transcriptional activity (Figure 3). In the nucleus, IKKα enhances p53-mediated GADD45 and BAD gene expressions by phosphorylating p53 at Ser20 [53] and stabilizing p53 protein levels [54], leading to the induction of apoptosis in response to ROS exposure. In response to DNA damage, both p53 and its homolog p73 function against NF-κB in deciding cell fate. Treatment with cisplatin induces IKKα nuclear translocation in human osteosarcoma-derived U2OS cells and hepatocellular carcinoma HepG2 cells in an ATM-dependent manner [23,55]. As shown in Figure 3, nuclear IKKα also stabilizes p73 protein through physical interaction in response to cisplatin [23]. Although the exact residue of p73 phosphorylated by IKKα has not yet been identified, a study showed that nuclear IKKα phosphorylates p73 within its N-terminal region, which may protect p73 from ubiquitination and proteasomal degradation [23]. These findings suggest an indispensable role of IKKα in cisplatin sensitivity. Different from ROS-induced nuclear IKKα on p53 stability in ROS-treated human MOLT-4 and HL-60 leukemia cells, cisplatin-activated IKKα nuclear translocation did not lead to p53 stabilization in HepG2 cells [53,54], suggesting that IKKα-dependent protein stabilization is cell type- and stimulus-specific.

### Nuclear IKKα is essential for cell cycle arrest and differentiation of keratinocyte in the epidermis and the morphogenesis of skeletal and craniofacial morphogenesis

**Figure 4:**
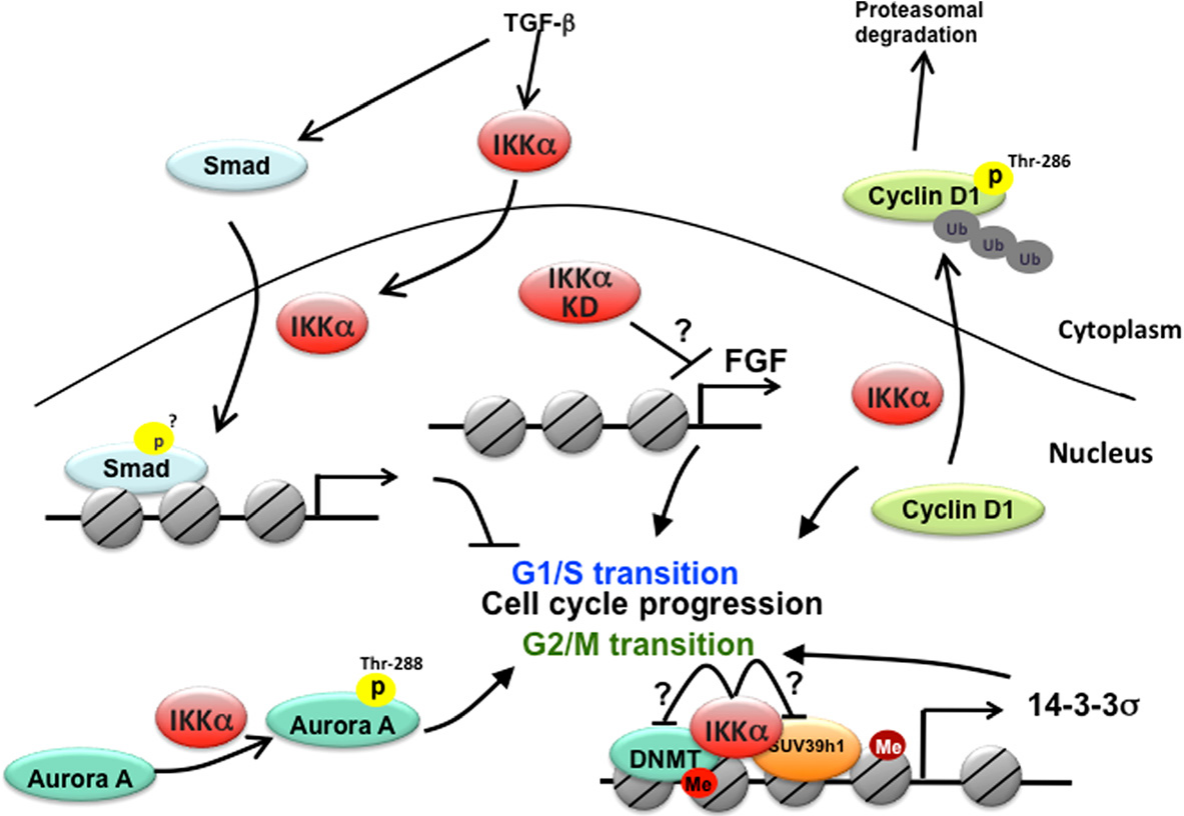
**Regulations of cell cycle progression by nuclear IKKα.** In the nucleus, IKKα is involved in cell cycle arrest at G1/S transition by increasing Smad transcriptional activity, facilitating cyclin D1 proteasomal degradation, and FGF gene expression. Nuclear IKKα also promotes G2/M phase progression by increasing kinase activity of Aurora A and by de-repressing 14-3-3 σ gene expression through preventing DNA and histone methylation on the promoter.

Unlike its well-established role in anti-apoptosis, the involvement of NF-κB in regulating cell cycle progression remains unclear. NF-κB activation is required for cell cycling in fibroblast [56], regenerating liver cells [57], breast cancer cells [58], and HeLa cells whereas NF-κB inhibition impairs cell cycle progression and retardation of G1/S transition [59]. During cell cycle, D-type cyclins (cyclin D1, D2, and D3) are critical for G1 to S phase progression. By phosphorylating the retinoblastoma tumor suppressor protein (pRb), cyclin D with its partner cyclin-dependent kinases (CDKs), releases the E2F family of transcription factors to activate the expression of cyclin E and several other genes required for the cell cycle progression [60,61]. The direct binding of NF-κB on the promoter region of cyclin D1 gene and the pronounced reduction of cyclin D1 expression by NF-κB inhibition provide additional evidence for the involvement of cyclin D1 transcription in NF-κB-mediated cell cycle progression [62-64]. However, several studies showed contradictory results in which NF-κB activation by overexpression of p65 or c-Rel causes cell-cycle arrest and induces cells to commit to terminal differentiation [65,66]. For instance, the G1-arrest by p65 occurs in pro-B but not in mature B cells [66], suggesting that this event depends on cell developmental stage. Interestingly, mouse with IKKα gene inactivation also had an unexpected excessive proliferation of the skin basal layer due to the absence of epidermal differentiation [67,68]. The specific roles of NF-κB in IKKα-mediated cell cycle arrest and subsequent differentiation in keratinocyte was initially proposed based on the observation that NF-κB activation is not detectable in keratinocytes from IKKα-null mouse skin [69]. However, a subsequent study pointed out that IKKα controls epidermal keratinocyte differentiation independently of NF-κB activation but regulates cyclin D1 protein stability in which cyclin D1 is overexpressed and predominantly localized in the nucleus of IKKα−/− MEF cells compared with parental MEF cells [70]. *In vitro* binding and kinase assays showed that IKKα directly binds cyclin D1 and phosphorylates it at Thr286. The cytoplasmic expression and increased degradation of cyclin D1 by reconstitution of IKKα in knockout cells further suggest that this phosphorylation by IKKα is required for nuclear export and turnover of cyclin D1 [71]. The predominantly nuclear localized cyclin D1 implies that IKKα may phosphorylate cyclin D1 in the nucleus and regulates its nuclear export. The potential nuclear function of IKKα in facilitating cyclin D1 protein degradation but not in NF-κB activation may be attributed to IKKα-mediated cell cycle arrest and differentiation of keratinocytes (Figure 4). Indeed, keratinocyte differentiation is associated with increased nuclear distribution of IKKα. Inactivation of the NLS by site-directed mutagenesis prevents IKKα from entering the nucleus without affecting its kinase activity and blocks the IKKα-induced differentiation of primary cultured IKKα^−/−^ keratinocyte, supporting an essential role of nuclear IKKα in the keratinocyte differentiation [8]. Likewise, Marinari *et al.* also found that nuclear IKKα can act as a tumor suppressor in stratified epithelia [72]. After stimulation with TGFβ, IKKα accumulates in the nucleus of keratinocytes and occupies the promoter of genes responsive to TGFβ-SMAD signaling to mediate TGFβ-induced Ovol1 and Mad1 upregulation and Myc downregulation (Figure 4). Such activity of nuclear IKKα is important for the anti-proliferative TGFβ pathway. In contrast, the expression and nuclear localization of IKKα are gradually reduced during malignant progression of squamous cell carcinoma (SCC) and acquisition of an invasive phenotype [72], which supports the tumor suppressive role of nuclear IKKα. However, the function of TGFβ-induced nuclear IKKα seems to counter its metastatic role in breast cancer cells [73] (please see below), suggesting a keratinocyte-specific role of nuclear IKKα in suppressing cell proliferation.

Besides the failure of epidermal differentiation, IKKα-deficient mice also exhibit abnormalities in skeletal and craniofacial morphogenesis [70,74], which is not observed in mice with systemic inhibition of NF-κB [75]. The results from these studies further support the dispensable role of NF-κB in nuclear IKKα-mediated keratinocyte differentiation. By introducing an epidermal-specific IKKα transgene into IKKα-deficient mice, most of these morphological abnormalities were completely rescued, suggesting that nuclear IKKα-dependent epidermal differentiation control skeletal and craniofacial morphogenesis [8]. In addition to targeting cyclin D1 protein degradation, another potential mechanism by which nuclear IKKα affects keratinocyte differentiation and craniofacial and skeletal morphogenesis is through repression of the fibroblast growth factor (FGF) family members [8], which bind to FGF receptor (FGFR) to antagonize bone morphogenic protein (BMP) signaling [76]. Since reintroduction of a catalytically inactive form of IKKα in IKKα−/− mice is still able to rescue epidermal differentiation and skeletal morphogenesis, the developmental functions of IKKα have been proposed to be independent of its protein kinase activity [76]. Therefore, nuclear IKKα may also contribute to the suppression of FGF transcription through a kinase-independent manner, hence excluding its involvement in phosphorylating histone H3 (Figure 4). Exploration of the kinase-independent roles of nuclear IKKα awaits further studies.

In addition to G1/S transition, IKKα also has a role in regulating the M phase of cell cycle as shown in Figure 4. Progression through the M phase of cell cycle is dependent on several mitotic kinases, including those of the Aurora families [77]. Aurora A localizes to the centrosome and functions in centrosome maturation and separation [77], and knock down of Aurora kinase A by siRNA increased the percentage of mitotic cells with high levels of Plk1 and cyclin B1 [78]. In a similar pattern to Aurora A siRNA knockdown, Prajapati *et al.* showed that silencing of IKKα but not IKKβ by siRNA also increased the number of HeLa cells at the G2/M phase and the levels of Plk1 and cyclin B1 [79]. These results further revealed that IKKα is associated with Aurora A in the centrosome and directly phosphorylates Aurora A at Thr288 [79], suggesting a nuclear function of IKKα in regulating the M phase of the cell cycle through Aurora A phosphorylation. In addition to targeting Aurora A, chromatin-bound IKKα also maintains the progression of G2/M phase during the cell cycle by preventing the silencing of 14-3-3σ, a check point protein for G2/M phase transition [80]. In IKKα-deficient keratinocytes that showed cell cycle arrest at the G2/M phase, the SUV39h1 histone trimethyltransferase and the Dnmt3a DNA methyltransferase were found to associate and methylate histone H3 lysine-9 (K9) and *14-3-3σ locus* DNA, respectively, which then silenced 14-3-3σ expression. Reintroduction of wild-type (WT) IKKα, but not its chromatin-unbound mutants bearing defects within the leucine zipper domain and helix-loop-helix motif, restored the 14-3-3σ expression by preventing the association of SUV39h1 and Dnmt3a with the 14-3-3σ locus, indicating that chromatin-associated IKKα prevents 14-3-3σ from hypermethylation (Figure 4). Interestingly, the kinase activity of IKKα is dispensable for blocking 14-3-3σ hypermethylation [80], suggesting that nuclear IKKα may protect 14-3-3σ from hypermethylation through an unexplored kinase-independent mechanism.

### Nuclear function of IKKα in tumorigenesis and metastasis

**Figure 5:**
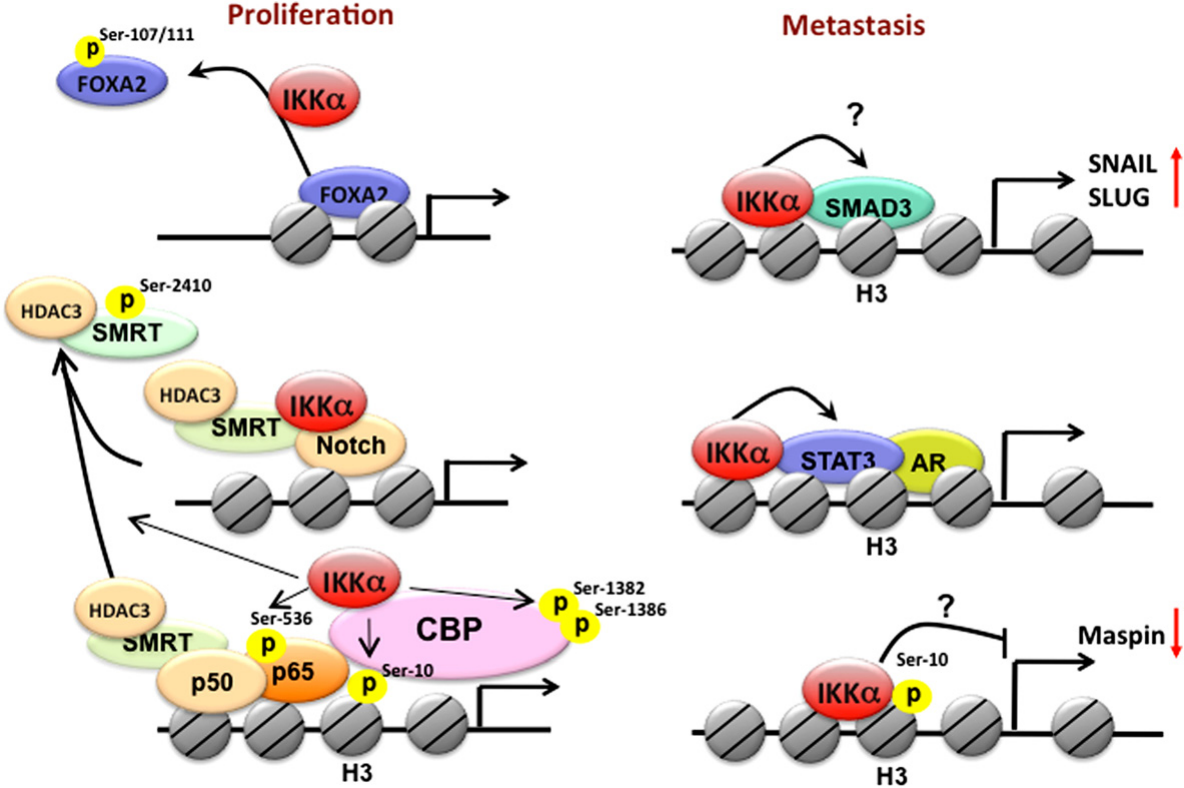
**Nuclear IKKα and tumor progression.** Nuclear IKKα promotes tumor growth by enhancing NF-κB- and Notch-dependent gene transcriptions and suppressing FOXA2-mediated gene expression. By promoting Smad and STAT3 transcriptional activity and suppressing maspin gene expression, nuclear IKKα contributes to cancer metastasis.

Constitutive activation of NF-κB has been found in many types of tumor cells. Most of these studies report an increased IKK activity that results in phosphorylation of IκBα; however, some have found little or no changes in the subcellular localization of p65 in some of the tumor cells. For instance, Fernández-Majada *et al.* reported that the increased IKK activity in colorectal cancer (CRC) cell lines and primary CRC is concomitant with undetectable levels of nuclear p65 and p52, which is consistent with the absence of p65 and p52 on different promoters of NFκB-target genes detected by ChIP analysis. These results indicate that NFκB activation may not be the main consequence of IKK activity in colorectal tumors, reflecting the substrate specificity of different IKK complexes. By immunohistochemistry staining and subcellular fractionation followed by Western blot analysis, they also showed that IKKα is present in the nucleus of most primary colorectal tumor tissues and CRC cell lines but not in HS27 or HEK-293 control cells. The increase in nuclear IKK activity in colorectal tumors is significantly correlated with SMRT phosphorylation at Ser2410 and its cytoplasmic translocation (Figure 5). At the chromatin level, the association of IKKα to specific Notch target promoters results in the release of chromatin-bound SMRT and thus activating *hes1, hes5,* or *herp2/hrt1* transcription, which promotes cell proliferation by repressing transcription of the cyclin-dependent kinase inhibitor p27^Kip1^[17]. In addition, we reported that enhancement of Notch transactivation by IKKα through inhibition of FOXA2/NUMB signaling is also likely to contribute to inflammation-mediated liver cancer progression [47]. Our other study also indicated that constitutively activated IKKα, found in certain human cancers, including lung, liver, pancreatic, and ovarian cancers, can phosphorylate and direct CBP to bind preferentially to NF-κB but not p53, thereby favoring proliferation and survival over p53-dependent apoptosis [15].

The epithelial to mesenchymal transition (EMT) is a crucial step in tumor progression in many tumor types. Independently of NF-κB activation, nuclear IKKα has been implicated in EMT by enhancing gene expression of SNAIL and SLUG transcription factors to downregulate expression of the adherens junction protein E-cadherin. As shown in Figure 5, IKKα enters the nucleus and regulates gene expression of SNAIL and SLUG by interacting with SMAD3 and controlling SMAD complex formation on the promoters of these two transcription factors in response to TGFβ activation, leading to metastasis of breast cancer cells [73]. Tumor-infiltrating immune cells expressing lymphotoxin-β [81] and RANKL [24] have also been found to induce activation and nuclear localization of IKKα in prostatic epithelial tumor cells (Figure 5). After castration, activated STAT3 has been reported to promote the transcriptional activity of unliganded androgen receptor in prostate cancer cells [82]. Lymphotoxin β-induced nuclear IKKα, in conjunction with STAT3, contributes to the emergence of castration resistance and enhances hormone-free survival and metastasis of prostate cancer by an NF-κB-independent, cell autonomous mechanism [81]. By targeting histone H3 Ser10 on the promoter of maspin, nuclear IKKα was also proposed to mediate the repression of maspin, a critical suppressor of metastasis, through an unidentified mechanism, which then commits malignant prostatic epithelial cells to a metastatic fate [24]. Similarly, we found that overexpression of HBx reduced maspin expression in Hep3B cells, and expression of wild-type IKKα but not its NLS mutant suppressed maspin expression in Hep3B cells, indicating that nuclear IKKα likely plays a role in HBx-mediated cell migration and invasion via suppressing maspin expression [14]. Although nuclear IKKα has been proposed to suppress maspin expression via histone H3 Ser10 phosphorylation [24] (Figure 5), it is still unclear how this histone phosphorylation reduces the promoter activity of maspin. Taken together, these findings indicate that a specific set of genes regulated by nuclear IKKα plays a critical role in tumorigenesis and metastasis. The detailed molecular mechanisms await further investigations.

### Potential mechanisms of IKKα nuclear translocation

Exploration of various nuclear IKKα functions raised a fundamental question of how IKKα travels from the cytoplasm to the nucleus. It is believed that, for a majority of proteins, NLS-bearing molecules are transported into the nucleus by forming a complex with importin α/β [83] or importin β alone [84]. A lysine-rich motif, Lys235, Lys236, and Lys237 within the kinase domain of IKKα has been shown to contain the NLS. Mutation of these residues attenuated the spontaneous nuclear import of IKKα but did not interfere with its kinase activity or binding to IKKγ [8]. We also characterized the signaling peptide for IKKα nucleo-cytoplasmic shuttling in response to HBx overexpression and found that in addition to these three lysines, two additional lysines (233 and 240) are also required for the nuclear translocation of IKKα [14]. The energy for the importin-based nuclear transport is provided by the small Ras family GTPase, Ran [85]. A dominant negative mutant of Ran has been reported to inhibit IKKα nuclear translocation [25], suggesting that the nuclear import of IKKα requires importins. However, the specific molecules that are involved in NLS-mediated IKKα nuclear translocation remain to be investigated.

Likewise, nuclear export signals (NES), which are recognized by a soluble export receptor (also known as Exportin 1 or CRM1), mediate nuclear export [86]. A study in 2002 by Birbach *et al.* showed that presence and incubation of LMB, an inhibitor of CRM1, enhanced the levels of IKKα in the nucleus [13]. This raises the possibility that IKKα can shuttle out of the nucleus through the CRM1 pathway and contains an NES to allow for the recognition and binding of CRM1 receptor. Based on the consensus NES sequence, which typically has a leucine-rich consensus sequence in the form of LX_1-3_LX_2-3_LXL (L=leucine and X=any amino acid) [86], there are two putative NESs (Leu601 ~ Leu612 and Leu714~Leu724) located at the C-terminus of IKKα. Leucine or isoleucine substitution within the motif containing residues 714–724 enhanced nuclear accumulation of IKKα, thereby supporting the presence of an NES for IKKα nuclear export [14].

**Figure 6:**
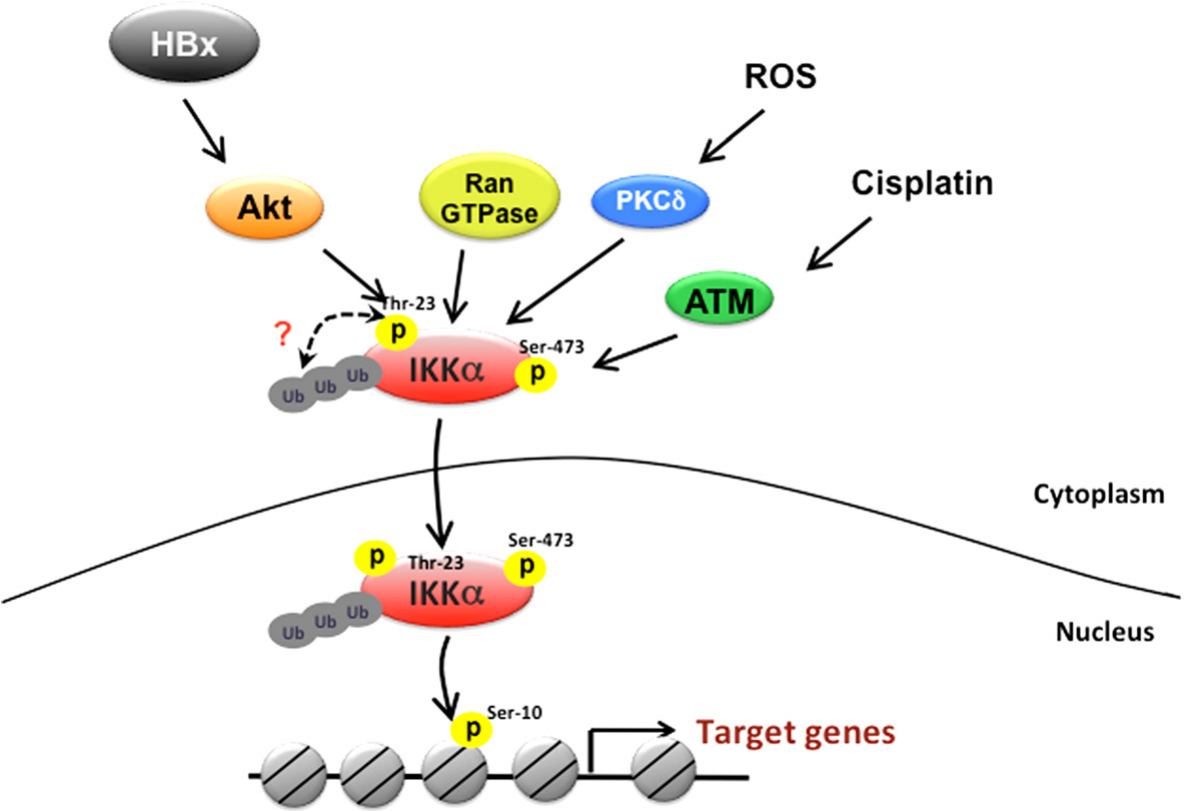
**Molecular mechanisms of IKKα nuclear transportation.** Ran GTPase activity is required for the nuclear transport of IKKα through interacting with importin-α. In response to HBx overexpression and cisplatin treatment, phosphorylations of IKKα at Thr23 and Ser473 by Akt and ATM respectively promote its nuclear translocation. The ubiquitination of IKKα is essential for the Akt-regulated IKKα nuclear import. Under exposure to ROS, activated PKCδ also enhances the nuclear accumulation of IKKα.

Since IKKα can enter the nucleus in response to diverse stimuli, including TNF-α [12,16], *Helicobacter pylori*[13], estrogen [20], EGF [45], and cisplatin [55], it is likely that signaling pathways, in addition to NLS and NES, are also critical for regulating IKKα nucleo-cytoplasmic shuttling (Figure 6). A kinase-dead mutant of IKKα (IKKα-K44M) has been shown to have lower nuclear accumulation than the wild type form, indicating that kinase activation is required for IKKα to translocate into the nucleus [13]. Activation of IKK complex usually involves trans-autophosphorylation by the catalytic domains of IKKα and IKKβ. However, knockdown of IKKβ by siRNA had no effect on *H. pylori*-induced IKKα nuclear translocation [22]. This suggests that IKKα can bypass the classic IKK complex activation pathway to enter the nucleus. As IKKβ does not exist in the nucleus, the distinct mechanisms by which the kinases are regulated may have a role in controlling the nuclear translocation of IKKα. Indeed, Akt, a mitogen-activated survival factor, has been shown to increase the activity of IKKα but not IKKβ by phosphorylating it at Thr23 in response to TNF-α [87]. Intriguingly, the signals that stimulate IKKα nuclear import, including HBx, EGF, HER2, and TNF-α, also commonly induce Akt activation. Akt-enhanced nuclear expression of IKKα is further augmented by overexpression of ubiquitin, suggesting that ubiquitination plays a role in Akt-regulated IKKα nuclear transportation [14]. Further investigations are necessary to identify the E3 ligase and the ubiquitination sites of IKKα.

In response to cisplatin-induced DNA damage, ATM has been shown to activate and phosphorylate IKKα at Ser473 in an *in vitro* kinase assay. Treatment with ATM inhibitors blocked the nuclear IKKα accumulation by cisplatin, suggesting that ATM plays a role in the nuclear translocation of IKKα. In addition, the active form of ATM was shown to colocalize with IKKα in the nucleus to mediate cisplatin-induced p73 protein stabilization and apoptosis [55]. These findings suggest that Ser473 phosphorylation by ATM may be a critical posttranslational modification for IKKα nuclear import and functions in response to cisplatin treatment. Similarly, in response to ROS exposure, PKCδ has also been demonstrated to increase the kinase activity and nuclear translocation of IKKα through protein-protein interaction (Figure 6). PKC-activated nuclear IKKα promotes the stability of p53 protein and mediates ROS-induced apoptosis [53]. However, the phosphorylation site mediated by ROS-activated PKC remains unclear. It would be of interest to further address whether phosphorylation of IKKα by Akt, ATM, or PKCδ at different residues affects its substrate preference in the nucleus.

## Conclusion

Since the first observation of nuclear localization of IKKα more than a decade ago, the field has gained tremendous insight into the distinct regulation and functions of nuclear IKKα. Other than IκB protein in the cytoplasm, these studies added histone and transcriptional co-factors as nuclear targets of IKKα for activation of NF-κB-dependent transcription. By targeting a growing list of substrates in the nucleus, IKKα has also been implicated in a variety of biological functions, including apoptosis, tumor suppression, immune functions, cell proliferation, and chromatin remodeling in an NF-κB-independent manner. Dysregulation of nuclear IKKα has been further linked to diabetes [88]. Contextual conditioned fear memory may also transduce IKKα to the nucleus of hippocampus for transcriptional regulation after memory recall [89]. These findings uncovered functional diversity of nuclear IKKα and other probable roles worthy of further exploration. For example, the involvement of nuclear IKKα in the termination of NF-κB signaling is an attractive yet under-developed area. The exploration of the nuclear role of IKKα in terminating NF-κB activity could lead us to understand how inflammation is resolved. The kinase-independent function of nuclear IKKα, which has been shown to control FGF suppression during epidermal differentiation and skeletal morphogenesis, is also another interesting area requiring further investigations. Addressing the kinase-independent functions of IKKα will likely provide more comprehensive explanation for the distinct roles between IKKα and its homologue, IKKβ.

In the past decade, there has been increased interest in the therapeutic disruption of the IKK/NF-κB pathway by using various approaches ranging from genetic manipulation to the development of pharmacologic inhibitors of IKK for inflammatory and autoimmune diseases [90,91]. Preclinical studies have also suggested IKKα/β as a therapeutic target for inhibition of NF-κB activity in various types of cancer [91], but translation of this mechanistic knowledge to clinically relevant therapeutic is much more difficult than researchers’ expectation. Instead, much of the effort toward the development of IKKβ and other NF-κB inhibitors has come from the pharmaceutical industry [90]. The limitation of clinical development of IKKβ inhibitors is probably due to the detrimental effects of excessive and prolonged NF-κB inhibition by IKKβ inhibition on innate immunity [92]. Although potent IKKα-specific inhibitors have not yet been described, the dispensability of IKKα in classic NF-κB activation and the unique roles of nuclear IKKα in modulating NF-κB-independent pathological activity, which are important for tumor progression, indicate that the chromatin-associated IKKα might be a promising target for therapeutic intervention in cancer. An ideal inhibitor designed to abrogate nuclear IKKα functions involved in a particular disease is therefore anticipated to minimize systemic toxicity and avoid general suppression of innate immunity, and may provide a more specific and safer therapeutic efficacy for cancer therapy.

## Abbreviations

BMP: Bone morphogenic protein; CBP: CREB-binding protein; CDK: Cyclin-dependent kinase; CRC: Colorectal cancer; CSC: Squamous cell carcinoma; CSE: Cigarette smoke extract; EMT: Epithelial to mesenchymal transition; ER: Estrogen receptor; ERE: Estrogen-responsive element; FGF: Fibroblast growth factor; GBS: Gram-positive human pathogen group B Streptococcus; HBx: Hepatitis B virus-encoded X protein; HDAC: Histone deacetylase; IKK: IκB kinase; KSHV: Kaposi’s sarcoma-associated herpesvirus; LMB: Leptomycin B; LPS: Lipopolysarccharide; MEF: Mouse embryonic fibroblast; NES: Nuclear export signal; NF-κB: Nuclear factor kappa B; NIK: NF-κB-inducing kinase; NLS: Nuclear localization sequence; PIAS1: Protein inhibitor of activated STAT1; pRB: Retinoblastoma tumor suppressor protein; ROS: Reactive oxygen species; TLR: Toll-like receptor; TNF-α: Tumor-necrosis factor alpha.

## Competing interests

These authors declare no competing conflict.

## Authors’ contributions

WCH designed the concept, collected information, and prepared the manuscript and figures. WCH and MCH wrote the manuscript. All authors read and approved the final manuscript.
